# Humoral and T-cell mediated response after administration of mRNA vaccine BNT162b2 in frail populations

**DOI:** 10.1016/j.jvacx.2022.100246

**Published:** 2022-12-05

**Authors:** Roberta Campagna, Laura Mazzuti, Giuliana Guerrizio, Chiara Nonne, Giuseppe Migliara, Corrado De Vito, Ivano Mezzaroma, Sabina Chiaretti, Caterina Fimiani, Valentina Pistolesi, Santo Morabito, Ombretta Turriziani

**Affiliations:** a Department of Molecular Medicine Sapienza University of Rome, Viale dell’Università, 33, 000185 Rome, Italy; b Department of Public Health and Infectious Diseases, Sapienza University of Rome, Piazzale Aldo Moro, 5, 00185 Rome, Italy; c Department of Translational and Precision Medicine, Sapienza University of Rome, Viale dell'Università, 37, 00185 Rome, Italy; d Department of Internal Medicine, Endocrine-Metabolic Sciences and Infectious Disease, Policlinico Umberto I, 155, 00161, Italy; e Department of Internal Medicine and Medical Specialties Sapienza University of Rome, Policlinico, 155, 00161 Rome, Italy

**Keywords:** Vaccine, SARS-CoV-2, HIV, Hemodialysis, Humoral response, T-cell response

## Abstract

Patients with frailty are considered to be at greater risk to get severe infection from SARS-CoV-2. One of the most effective strategies is vaccination.

In our study we evaluated both the humoral immune response elicited by the vaccination at different time points, and the *T*-cell response in terms of interferon (IFN)-γ production in frail patients and healthy donors.

Fifty-seven patients (31 patients undergoing hemodialysis and 26 HIV positive subjects) and 39 healthcare workers were enrolled. All participants received two doses of the mRNA vaccine BNT162b2.

Healthcare workers showed a significantly higher antibody titer than patients twenty-one days after the first dose (p < 0.001). From the same time point we observed for both groups a decay of the antibody levels with a steeper slope of decline in the patients group. Regarding *T*-cell response the only significant difference between non-reactive and reactive subjects was found in median antibody levels, higher in the responders group than in non-responders.

The healthcare workers seem to better respond to the vaccination in terms of antibodies production; the lack of *T*-cell response in about 50% of the participants seems to suggest that in our study population both humoral and cell-mediated response decline over time remarking the importance of the booster doses, particularly for frail patients.

## Introduction

At the end of 2019, a novel coronavirus, later named severe acute respiratory syndrome coronavirus 2 (SARS-CoV-2) emerged in the city of Wuhan, in Hubei province in China, causing the coronavirus disease 2019 (COVID-19) responsible for the pandemic state [1].

Coronaviruses can infect several avian and mammalian hosts [2]. Most coronaviruses that are pathogenic to humans only cause mild illnesses [3] exception made for severe acute respiratory syndrome coronavirus (SARS-CoV) [4] and Middle East respiratory syndrome coronavirus (MERS-CoV) [5], [6] responsible for two major epidemic outbreaks of the 21st century. People infected with SARS-CoV-2 show a wide range of symptoms among which general malaise, cough and fever [7]. In addition to that, the most severe cases can be characterized by acute respiratory distress syndrome (ARDS) and acute lung injury which leads to inflammation, damage of the alveolar lumen and pneumonia with possible fatal outcome[8], [9].

Patients with frailty are at a greater risk to get severe infection which can require hospitalization and lead to poor outcome [10], [11]. In particular patients with kidney disease may experience an immune system dysregulation that makes them more susceptible to infections [12]. Not only patients receiving in-centre dialysis can be more exposed to SARS-CoV-2 infection [13] but also chronic kidney disease (CKD) has been found to be a risk factor for severe COVID-19 and mortality[14], [15], [16], [17]. Different works also seem to demonstrate that people living with HIV (PLWH) having a dysregulated immune response could be at greater risk to develop severe COVID-19, and that’s especially true for those who experienced previous pulmonary events [18], [19]. Despite the contrasting results from different groups [20], WHO considers HIV a significant risk factor for developing critical illness [21].

Even though the use of antiviral drugs such as remdesivir [22] and treatment with monoclonal antibodies have been approved [23] the most effective strategy to limit the viral spread and to protect most vulnerable patients remains, at present, vaccination which enables the activation of all the components of the adaptative immune system. The first authorized vaccine to treat SARS-CoV-2 infection has been the BNT162b2 (Pfizer–BioNTech), an mRNA-based vaccine encoding for the spike protein, the expression of which elicits the activation of the immune response [24]. On December 2020 Italy started the vaccination campaign first involving healthcare workers, followed by some categories of patients with frailty.

The aim of our study was to evaluate the kinetic of the antibody response and the *T*-cell mediated response, in terms of interferon (IFN)-γ production, after the administration of two doses of the Pfizer–BioNTech mRNA vaccine in frail patients and healthy donors.

## Methods

### Study population

For this study 58 patients and 69 healthcare workers (HCWs) were enrolled. Twenty-six HIV-1 infected subjects, followed in an out-patient basis at the Department of Internal Medicine and Infectious diseases, were included. The following information were extracted from the medical records: demographics (age, gender), time from HIV-1 diagnosis (years), time of ARV exposure (years), CDC classification stage, current ARV, hepatitis C virus (HCV) co-infection, HIV-RNA level, CD4^+^ T cells nadir, current CD4 + T cell % and absolute count. Moreover, the presence of co-morbidities (including diabetes, hypertension, cardiovascular diseases (CVD), dyslipidemia) was recorded (Table 1).

**Table 1:** Demographic and clinical features of HIV patients.

HIV patients, n	26
Male gender, n, %	19 (73 %)
Age, years, median, IQR	65 (58–73)
HIV+, years, median, IQR	28 (20–32)
TARV, years, median, IQR	25 (20–28)
CD4 + NADIR, median, IQR	87 (42–249)
CD4+%, median, IQR	28 (20–38)
CD4 + absolute, median, IQR	639 (451–786)
HCV, n, %	5 (21 %)
Co-morbidities, n, %	15 (58 %)
*Diabetes*, n, %	3 (20 %)
*Dyslipidemia*, n, %	8 (53 %)
*CVD*, n, %	4 (27 %)
*Hypertension*, n, %	7 (47 %)

Thirty-two patients undergoing maintenance hemodialysis (MHD) for end stage chronic kidney disease (ESKD) were included. Demographic and clinical features of hemodialysis patients are reported in Table 2.

**Table 2:** Demographic and clinical features of hemodialysis patients.

Maintenance hemodialysis patients, n	32
Age, years, median, IQR	64 (55–78)
Male gender, n, %	20 (62.5 %)
Body Mass Index, Kg/m^2^, median, IQR	22.4 (20.4–25.1)
Hemodialysis vintage, months, median, IQR	47 (23–98)
Previous kidney transplant, n, %	6 (18.7 %)
Vascular Access	
*Arteriovenous fistula, n, %*	26 (81.3 %)
*Tunnelled central venous catheter, n, %*	6 (18.7 %)
Dialysis frequency	
*Thrice weekly, n, %*	26 (81.3 %)
*Twice weekly, n, %*	6 (18.7 %)
Dialysis modality	
*Bicarbonate dialysis, n, %*	20 (62.5 %)
*On-line hemodiafiltration, n, %*	12 (37.5 %)

All participants received two doses of the mRNA vaccine BNT162b2 produced by Pfizer-BioNTech.

To analyze the kinetic of the antibody response, sera samples were collected 7 and 21 days after receiving the first dose, and 7, 14, 21, 90 and 270 days after the second dose.

Samples were centrifuged for serum separation and stored at −20° C until analysis. Sera were tested using the LIAISON® SARS-CoV-2 TrimericS IgG kit (DiaSorin S.p.A., Saluggia, Italy) an indirect chemiluminescence immunoassay (CLIA) technology for the detection of serum IgG antibodies to SARS-CoV-2 trimeric spike protein. IgG titers were expressed in Binding Antibody Units/ml (BAU/ml), the assay quantification range is 4.81 to 2080 BAU/ml and the cut-off value is 33.8 BAU/ml. For a sub-group/-set of this population we were also able to study the cell-mediated response. Specifically, 29 patients undergoing hemodialysis and 23 HCWs were included. For each participant a plasma sample was collected 270 days after receiving second dose; the samples were analysed to evaluate the production of IFN-γ by *T*-cells after stimulation with different peptides using the IFN-gamma release assay Covi-FERON test by SD biosensor.

The study was granted ethical approval by the local ethical committee, protocol number 0486/2021.

### Statistical analysis

Descriptive statistics were reported using median and interquartile ranges for continuous variables and using absolute and relative frequencies for dichotomous variables. Univariable analysis was performed using the Wilcoxon rank-sum test to compare continuous variables between patients and HCWs, whereas Pearson’s chi-squared test or Fisher’s exact test was used for dichotomous and categorical variables, as appropriate. A multivariable generalized estimating equation population-averaged regression model with an identity link, a gaussian error structure and exchangeable correlation structure was built to estimate beta coefficients (β) and associated confidence intervals (CI) of factors influencing IgG levels over time after the second dose of vaccine, considering the clustering within participant due to repeated measures [25]. Variables were included in the model based on expert opinion. The final model included the following variables: sex (0 = woman; 1 = man); age (continuous); HIV/Hemodialysis (0 = No; 1 = Yes); antibody levels 21 day after first dose of vaccine (continuous); time from second dose of vaccine (7 days (t7, ref.), 14 days (t14), 21 days (t21), 90 days (t90), 270 days (t270)); interaction term between HIV/Hemodialysis and time from second dose.

For the participants undergone Covi-FERON test a univariable analysis was performed to compare continuous and dichotomous variables between reactive and non-reactive subjects. Due to the small sample size, no multivariable analysis was performed.

All analyses were performed using STATA 17.0 (StataCorp LLC, 4905 Lakeway Drive, College Station, 322 Texas, USA) and SPSS version 27.0. A two-sided p-value < 0.05 was considered statistically significant.

## Results

A total of 57 HIV/Hemodialysis patients and 39 HCWs were enrolled in the study. Demographic characteristics of the two groups and antibody titration at sample collection time points are reported in table 1, with the statistical characteristics of the appropriate univariate test.

The median age for the HIV group was 65 years (IQR 58–73). In addition, 19/26 were males with a median of 28 years (IQR 20–32) from HIV-1 diagnosis. All the patients were under antiretroviral treatment from a median of 25 years (IQR 20–28). Thirteen subjects had a previous history of AIDS diagnosis, according with CDC classification. HIV-RNA plasma level at baseline was under the threshold of 37 copies/ml in all subjects. The immunological profile was represented by a median CD4 + T cell nadir of 87 (IQR 42–249) cells/μL, a median current CD4 + T cell count of 639 (IQR 451–786) cells/μL and a mean CD4 + T cell percentage of 28 (IQR 20–38). HCV co-infection was present in 5 enrolled participants. At least one non-communicable disease was presented by 15 PLWH: the most prevalent was dyslipidemia followed by hypertension, type 2 diabetes and cardiovascular diseases.

Overall, the vast majority of PLWH enrolled in the current study were receiving an INSTI-based regimen, followed by a DRVc-based therapy.

In the hemodialysis group 20 patients (62.5 %) were male; the median age was 64 (IQR 55–78). Median dialysis vintage was 47 months (IQR 23–98). Hemodialysis vascular access was arteriovenous fistula in 26 patients; the remaining 6 patients underwent dialysis treatment trough a tunnelled central venous catheter. In 26 patients (81.3 %) a thrice-weekly MHD was prescribed, while 6 patients received twice-weekly treatment. Dialysis modalities adopted were on-line hemodiafiltration (37.5 %) and bicarbonate dialysis (62.5 %). Anticoagulation of extracorporeal circuit was performed by using low molecular weight heparin (LMWH) in all patients. Six patients had a previous kidney transplant, but in any case, they started MHD at least 18 months prior to the study.

**Fig. 1:**
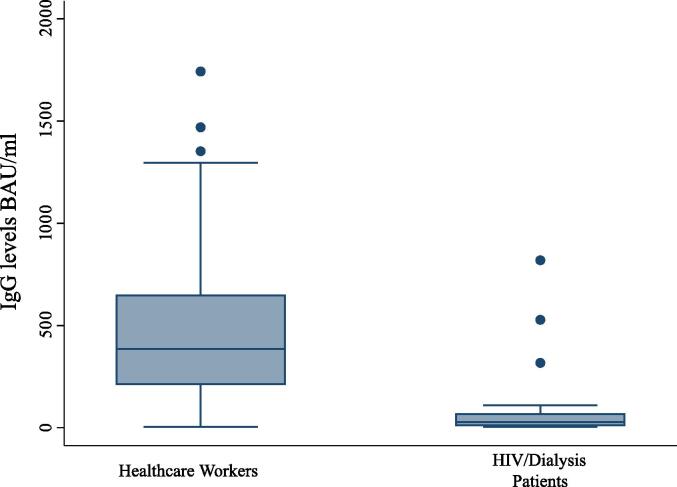
Box plot of IgG levels 21 days after first dose in healthcare workers and HIV/Dialysis Patients, showing medians, interquartile ranges and outliers.

**Fig. 2:**
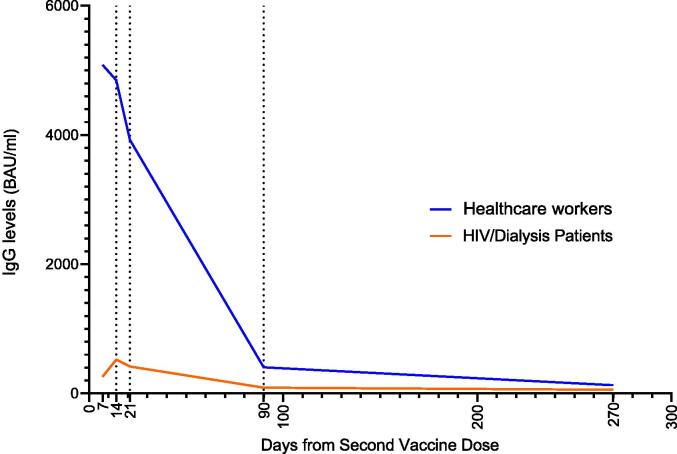
Trend of median IgG levels at 7, 14, 21, 90 and 270 day after the second vaccine dose for healthcare workers (blue line) and HIV/Hemodialysis patients (orange line). (For interpretation of the references to colour in this figure legend, the reader is referred to the web version of this article.)

Grouping the patients together we found that subjects in the HIV/Hemodialysis group were older (69 vs 46 years, p < 0.001) and more frequently males (69 % vs 25 %, p < 0.001) than HCWs. Twenty-one days after the first dose antibody levels were higher in the HCWs than in HIV/Hemodialysis patients (386.1 BAU/ml, IQR 210.6 – 651.3 vs 28.6, IQR 8.8 – 70.2, p < 0.001), see Fig. 1. This difference held true at each T after second dose, except for T270 (56, IQR 31.8–152 vs 128.5, IQR 84.1–203 BAU/ml, p = 0.052), see Fig. 2. The average number of IgG antibodies measurements per participants was 3.8. In Table 3 are reported the antibody levels (median, IQR) measured at all time points.

**Table 3:** Characteristics of participants enrolled in the study and antibody levels measured at the different time points.

	HIV/Hemodialysis	Healthcare Workers	p-value
**Participants,** n, %	39 (40.63 %)	57 (59.4 %)	
**Gender (male),** n, %	27 (69 %)	14 (25 %)	<0.001
**Age,** years, median, IQR	69 (58, 76)	46 (34, 59)	<0.001
**T21 I Dose,** BAU/ml**,** median, IQR	28.6 (8.814, 70.2) (n = 39)	386.1 (210.6, 651.3) (n = 57)	<0.001
**T7 II Dose,** BAU/ml, median, IQR	261.3 (56.94, 774.8) (n = 39)	5083 (3172, 7150) (n = 57)	<0.001
**T14 II Dose,** BAU/ml, median, IQR	525.2 (248.3, 1404) (n = 35)	4849 (3172, 6344) (n = 49)	<0.001
**T21 II Dose,** BAU/ml, median, IQR	418.6 (163.93, 781.95) (n = 36)	3926 (2262, 5694) (n = 49)	<0.001
**T90 II Dose,** BAU/ml, median, IQR	88.92 (39.78, 228.02) (n = 25)	405.6 (191.88, 1021.8) (n = 33)	<0.001
**T270 II Dose,** BAU/ml, median, IQR	56.4 (31.8, 152) (n = 21)	128.5 (84.11, 203) (n = 18)	0.052
HIV: Human Immunodeficiency Virus; BAU: Binding Antibody Unity; ml: Milliliters. n: Numbers of observation			

The regression model showed that both male gender (β 27.8, 95 % CI −538.44, 594.00, p = 0.923) and age (β −15.3, 95 % CI −35.95, 5.31, p = 0.146) didn’t influence IgG levels over time.

At the multivariable analysis, HIV/Hemodialysis patients had significant lower levels of IgG levels (β −3351.8, 95 %CI −4202.9 - −2500.7), while antibody levels 21 days after the first dose were slightly correlated with post-second dose levels (β 2.3, 95 %CI 1.5 – 3.1). Over time, the model showed a significant decrease of IgG with respect to the reference time (7 days after second dose) at t21 (β 997.2, 95 %CI 1520.5 – 473.9), t90 (β −4596.1, 95 %CI −5194.8 – −3997.4) and t270 (β −5079.0, 95 %CI −5834.0 – −4324.1), although for HIV/Hemodialysis patients it is greatly reduced (T14: β 1317.9, 95 %CI 509.7 – 2126.1;T90: β 4373.1, 95 %CI 3454.3 – 5291.8; T270: β 4798.6, 95 %CI 3740.9 – 5856.3). In Table 4 are reported the estimated beta coefficients (β) and associated confidence intervals (CI) of factors influencing IgG levels over time after the second dose of vaccine.

**Table 4:** Multivariable generalized estimating equation population-averaged regression model for IgG antibodies levels.

	β (95 % CI)	p-value
**Group**		
**Healthcare Workers**	Ref.	–
**HIV/Hemodialysis Patients**	−3351.78 (-4202.87, −2500.69)	<0.001
**Gender**		
**Female**	Ref.	–
**Male**	27.8 (-538.44, 594.00)	0.923
**Age (years)**	−15.3 (-35.95, 5.31)	0.146
**T after II dose, days**		
**T7**	Ref.	–
**T14**	−1530.42 (-676.46, 369.63)	0.565
**T21**	−997.21 (-1520.53, −473.89)	<0.001
**T90**	−4596.11 (-5194.82, −3997.41)	<0.001
**T270**	−5079.03 (-5833.97, −4324.09)	<0.001
**IgG at T21 after first dose, BAU/ml**	2.34 (1.55, 3.13)	<0.001
T*Group		
7*HIV/Hemodialysis Patients	Ref.	–
14* HIV/Hemodialysis Patients	676.07 (-136.99, 1489.14)	0.103
21* HIV/Hemodialysis Patients	1317.91 (509.66, 2126.16)	0.001
90*HIV/Hemodialysis Patients	4373.07 (3454.34, 5291.81)	<0.001
270*HIV/Hemodialysis Patients	4798.57 (3740.87, 5856.27)	<0.001
β: Beta Coefficient; CI: Confidence Interval; BAU: Binding Antibody Unity; Ref.: Reference; HIV: Human Immunodeficiency Virus.		

### Covi-FERON response

**Fig. 3:**
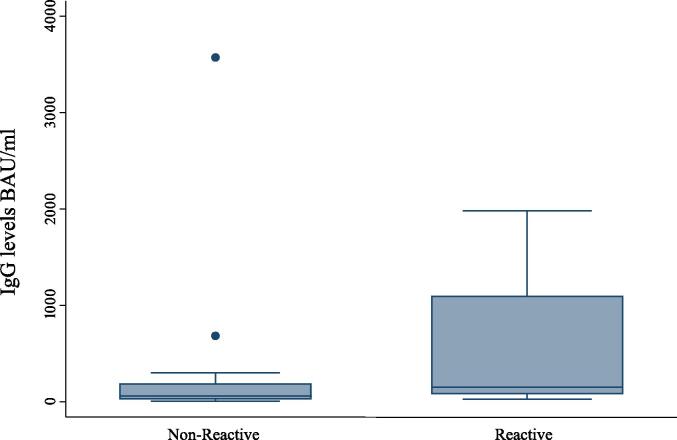
Box plot of IgG levels before the third vaccine dose in Non-Reactive (subjects in which no effector *T*-cell-mediated response was detected) and Reactive (subjects in which an effector *T*-cell-mediated response was detected) subjects, showing medians, interquartile ranges and outliers.

Fifty-one participated in the Covi-FERON analysis. Considering the two groups together we observed that 46 % of the subject responded to the test. Individuals in which no effector *T*-cell mediated response was detected were considered non-reactive, while those with a detectable effector *T*-cell mediated response were considered reactive. Patients and HCWs responded similarly. The only significant difference between non-reactive and reactive subjects was found in median antibody levels, higher in the responders group than in non-responders (177.5, IQR 81.7 – 1160, vs 61.4, IQR 23.4 – 189 BAU/ml, respectively), see Table 5 and Fig. 3.

**Table 5:** Characteristics of participants subset for the Covi-FERON analysis.

	Non-Reactive	Reactive	p-value
**Participants,** n, %	27 (52.9 %)	24 (47.1 %)	
**Group (Hemodialysis Patients),** n, %	14 (51.9 %)	15 (62.5 %)	0.44
**Gender (Male),** n, %	9 (33.4 %)	13 (61.9 %)	0.13
**Age,** years**,** median, IQR	57 (50, 72)	55.5 (38, 67)	0.47
**IgG, BAU/ml,** median, IQR	61.4 (23.4, 189)	177.5 (81.7, 1160)	0.009
HIV: Human Immunodeficiency Virus; BAU: Binding Antibody Unity; ml: Milliliters.

## Discussion

In our study we focused our attention on the ability of the BNT162b2 mRNA vaccine of inducing an immune response both in patients and healthy donors. We observed that both groups were able to mount an antibody response after receiving two doses of vaccine.

Twenty-one days after receiving the first dose we had the highest number of measurements and at that time point the seroconversion rate was different between patients and HCWs (50 % and 92 % respectively). The seroconversion rate reached its peak 14 days after the second dose for the patients group (91 %) while at the same time point 100 % of the HCWs showed seroconversion.

Using the antibody levels measured 7 days after the first dose as our baseline, we found that they were significantly higher in the HCWs group at all time points except for 270 days after the second dose. The individuals in the patient group were older than HCWs and more frequently males. To rule out the possibility that our results could have been confounded by those parameters a multivariable analysis was performed including gender and age as independent variables. Moreover, the model showed that neither age nor gender had an independent influence on the humoral response. Despite being effective in both populations, our results seem to suggest that the individuals presenting an underlying medical condition are less capable to develop and maintain a strong antibody response after vaccination.

Several studies show that most PLWH successfully build an efficient humoral response after the delivery of the BNT162b2 mRNA vaccine even though it is weaker if compared with immunocompetent individuals [26], [27]. Antinori *et al.* also found that in HIV-1 patients the antibody production is strongly related to the CD4 + T cell count at the time of vaccination, suggesting that the measurement of this parameter could be used to better adjust the vaccination strategy in this specific population. In contrast, in our study we didn’t observe any correlation between the antibody levels and the CD4 + T cell count at any time point (data not shown), probably due to the small number of patients. The decreased immunogenicity of the vaccine has also been demonstrated for ESKD patients that show a lower magnitude of the humoral response when compared to the general population with seroconversion rates varying from 17.4 to 96 % [28], [29].

Consistently with other data in literature, we observed for both groups a decline of the antibody levels some weeks after the administration of the second dose of vaccine [30], [31]. In our study population the decay started extremely early, 21 days after receiving the second vaccine dose, even though for the HIV/Hemodialysis patients the decay was greater. In a study from Anand *et al.* conducted on hemodialysis patients, they found that 20 % lost detectable IgG response within 6 months following vaccination. In the same study 56 participants had a breakthrough COVID-19 infection and among these, patients had lower peak and pre-breakthrough RBD IgG index values compared with controls [32]. A reduction of antibody levels following vaccination has been documented as well for HIV patients who received ChAdOx1 viral vector vaccine. However in this study population the antibody drop does not seem to be related to the HIV status but rather to the older age and the number of chronic conditions [33].

For what concerns the *T*-cell response, the only noteworthy aspect was the higher level of antibodies in those who showed an IFN- γ production. This observation seems to suggest that the preservation of the *T*-cell response could be linked to a stronger production of antibodies following vaccination. Unfortunately, in our study we were only able to measure *T*-cell response at t270, therefore it is not possible to establish with certainty if the cell mediated response wanes together with the antibody level. This result seems to be in contrast with what observed in people who recovered from COVID-19 where no correlation between antibody levels and *T*-cells was found [34], [35].

The low percentage of individuals able to produce a *T*-cell mediated response could be linked with the kind of test used since we were not able to define the different cell population or the different cytokines produced in response to vaccination, but only to evaluate the IFN- γ production.

The clinical trial of BNT162b2 vaccine and along with other studies proved that protection against the symptomatic disease exerted by T cells starts about 10 days after the administration of the first dose, while high levels of neutralizing antibodies are only detectable 21 days after the first dose, emphasizing that the humoral response is not the only being necessary for protection against viral infection [36], [37].

Furthermore, despite the waning of the antibody levels and the ability of SARS-CoV-2 variants to partially escape the humoral response, most people who receive two vaccine doses and are later infected with viral variants generally develop only mild symptoms. This could be explained by the presence of heterogeneous Spike-specific *T*-cells able to recognize different regions of the Spike protein [38], [39]. Unlike humoral response it is more difficult to quantify the magnitude of the cell-mediated immunity. Significant variations of the *T*-cell response were observed not only in particular categories, like people over 80 [40] and patients with immune system deficiencies [41] but also in individuals of similar age and without medical conditions [42]. In view of these considerations, it seems clear that further studies are necessary to fully understand the correlation between all the immune system components. The decay of the antibody levels and the weak *T*-cell response that we observed support the necessity of booster doses for both patients and healthy donors.

The main limitations that should be acknowledged in our study are its monocentric design, the small number of participants that were recruited, especially for the analysis of the *T*-cell response and the lack of plasma samples at different time points to ascertain if the production of IFN- γ follows a similar trend as the one observed for the humoral response.

Despite the limitations of our study, it seems undeniable the importance of vaccination to maintain a protection against SARS-CoV-2 infection especially in patients with frailty. Given these considerations we can affirm that monitoring the extent and the duration of the immune response in people considered at a higher risk of severe infection could help improving the immunization plan for those categories of patients.

Author contributions

All authors contributed to the study conception and design. Material preparation and analysis were performed by Roberta Campagna, Laura Mazzuti, Giuliana Guerrizio and Chiara Nonne. Statistical analysis was performed by Giuseppe Migliara and Corrado De Vito. Clinical data and samples were collected by Ivano Mezzaroma, Caterina Fimiani, Sabina Chiaretti, Santo Morabito and Valentina Pistolesi. The first draft of the manuscript was written by Roberta Campagna, and Ombretta Turriziani, and all authors commented on previous versions of the manuscript. All authors read and approved the final manuscript.

Funding

This work was supported by “Progetto di Ricerca di Ateneo” 2019.

## Declaration of Competing Interest

The authors declare that they have no known competing financial interests or personal relationships that could have appeared to influence the work reported in this paper.

## Data Availability

Data will be made available on request.
